# Linking Vascular Structure and Function: Image-Based Virtual Populations of the Retina

**DOI:** 10.1167/iovs.65.4.40

**Published:** 2024-04-29

**Authors:** Rémi J. Hernandez, Savita Madhusudhan, Yalin Zheng, Wahbi K. El-Bouri

**Affiliations:** 1 Liverpool Centre for Cardiovascular Science, University of Liverpool and Liverpool Heart and Chest Hospital, Liverpool, United Kingdom; 2 Department of Cardiovascular and Metabolic Medicine, Institute of Life Course and Medical Sciences, University of Liverpool, Liverpool, United Kingdom; 3 St Paul's Eye Unit, Liverpool University Hospitals NHS Foundation Trust, Liverpool, United Kingdom; 4 Department of Eye and Vision Sciences, Institute of Life Course and Medical Sciences, University of Liverpool, Liverpool, United Kingdom

**Keywords:** macula, computational model, sensitivity analysis, hemodynamics, digital twins, in silico model

## Abstract

**Purpose:**

This study explored the relationship among microvascular parameters as delineated by optical coherence tomography angiography (OCTA) and retinal perfusion. Here, we introduce a versatile framework to examine the interplay between the retinal vascular structure and function by generating virtual vasculatures from central retinal vessels to macular capillaries. Also, we have developed a hemodynamics model that evaluates the associations between vascular morphology and retinal perfusion.

**Methods:**

The generation of the vasculature is based on the distribution of four clinical parameters pertaining to the dimension and blood pressure of the central retinal vessels, constructive constrained optimization, and Voronoi diagrams. Arterial and venous trees are generated in the temporal retina and connected through three layers of capillaries at different depths in the macula. The correlations between total retinal blood flow and macular flow fraction and vascular morphology are derived as Spearman rank coefficients, and uncertainty from input parameters is quantified.

**Results:**

A virtual cohort of 200 healthy vasculatures was generated. Means and standard deviations for retinal blood flow and macular flow fraction were 20*.*80 ± 7*.*86 µL*/*min and 15*.*04% ± 5*.*42%, respectively. Retinal blood flow was correlated with vessel area density, vessel diameter index, fractal dimension, and vessel caliber index. The macular flow fraction was not correlated with any morphological metrics.

**Conclusions:**

The proposed framework is able to reproduce vascular networks in the macula that are morphologically and functionally similar to real vasculature. The framework provides quantitative insights into how macular perfusion can be affected by changes in vascular morphology delineated on OCTA.

The retina is a highly oxygen-dependent, complex band of tissue at the back of the eye that plays a key role in visual function. It requires close interplay among many different cell types and supporting structures, including a complex vascular system.^1^ As a result, the retina is sensitive to small changes that may lead to loss of visual functions. In silico modeling has the potential to offer insight into the complex interactions between the retinal environment and the underlying causes of retinal diseases. In particular, virtual populations and in silico clinical trials are a promising way to enhance basic research and clinical trials.^1^ Optical coherence tomography angiography (OCTA) is a non-invasive imaging modality that offers three-dimensional, high-resolution angiograms of the macula, which is the central-most area of the retina. Several microvascular metrics, such as vessel density and fractal dimension, have been suggested to quantify the quality of the microvasculature on OCTAs.^2^ Using those metrics, several microvascular changes have been linked not only with aging and diseased retinae^3^^–^^7^ but also with cerebrovascular changes and cardiovascular diseases.^8^^–^^10^ For the brain and heart vasculatures, virtual populations have been developed, but similar work is yet to be done for the retinal vasculature or for the linked cerebral–retinal vasculature.^1^

Alterations to the retinal and choroidal vasculature are expected to negatively affect the perfusion of the retina. Ischemia and hypoxia are likely involved in the pathogenesis of several retinal diseases, particularly in neovascular diseases such as neovascular age-related macular degeneration, proliferative diabetic retinopathy, or retinal vein occlusion, which are characterized by pathological growth of blood vessels through angiogenesis.^11^ Although angiogenesis and vascular endothelial growth factor (VEGF) are essential to the development of the vascular and in maintaining physiological conditions, the upregulation of VEGF is involved with the development of pathological neovasculature.^12^^,^^13^ Hypoxia is a known upregulating factor of VEGF, and targeting ischemia-induced angiogenic pathways might improve treatment outcomes.^14^

Retinal oximeters provide a way to analyze oxygen saturation in some of the larger blood vessels of the superficial retina. However, experimental measurements in the tissue and in the capillaries of the retina remain difficult or invasive, so there is still uncertainty regarding the role of hypoxia in the pathogenesis of neovascular diseases. Furthermore, it also remains unclear how changes in microvascular metrics computed on OCTAs relate to the quality of blood perfusion. Computational models of the retina and its vasculature, combined with mathematical models of hemodynamics and oxygen transport, can help to link vascular structure and function.

Retinal hemodynamics and oxygenation have received considerable attention from the modeling community in recent years.^1^^,^^15^^–^^24^ Compartmental models^15^^,^^18^^,^^21^ and/or symmetrical branching networks^18^^,^^22^^,^^23^ are used in many of these models. These approaches are favored for their simplicity and adaptability to systems with limited information; however, they fail to reproduce the complexity and heterogeneity of the retinal vasculature.^24^ In contrast, models based on vascular networks reconstructed from imaging data^16^^,^^20^ are more faithful to the morphology of the retina, but the reconstruction of the network is arduous; therefore, only a limited number of eyes can be modeled. Space-filling algorithms offer a way to circumvent these problems by generating heterogeneous networks with characteristics similar to those of real vasculature.^17^^,^^25^^,^^26^ For example, Causin et al.^17^ used diffusion-limited aggregation because it creates structures with a fractal dimension similar to that of retinal vasculature. The class of space-filling algorithms referred to as constrained constructive optimization (CCO) algorithms is another approach that includes rules and constraints meant to reproduce the angiogenesis process.^25^^,^^26^ It has been applied to the retinal vasculature^27^^,^^28^ but only to create synthetic data for deep-learning applications.

Vascular morphology has been established as a biomarker for the development, progression, and prognosis of several retinal diseases,^29^^,^^30^ including diabetic retinopathy^31^^,^^32^ and age-related macular degeneration.^6^^,^^33^ Changes in these metrics may indicate impairment to retinal or macular blood flow, which could contribute to development of the disease. However, quantifying these impairments in a sufficiently large population is challenging with conventional experimental techniques.

The modeling framework presented here can quantify these impairments in large virtual populations. The model captures the complexity of the macular vasculature and is able to link imaging biomarkers with physiological parameters. Our study sought to (1) develop a method for generating coherent vascular networks in the retina and macula, adaptable to virtual population generation; (2) propose a mathematical model of blood flow in the virtual vasculatures; and (3) quantify associations between macular vascular morphology and hemodynamics parameters in a healthy retinal population. All three capillary layers were modeled in the macula. We then compared the microvascular structure of the model with OCTA measurements and validated the blood flow model against two independent experimental studies. A global sensitivity analysis of the hyperparameters of the method is detailed, laying the foundation for generating tailored retinal populations based on the distribution of morphological metrics. The uncertainty quantification results then presented show the robustness of the predictions of our model. We also provide a discussion of the results and summarize the conclusions and future perspectives.

## Methods

In this section, we describe the proposed models, the model that generates the retinal vasculature from the macro- to microscale, and the proposed hemodynamic model.

### Structural Model

The structural model generates retinal vasculature from the macroscale (arteries, arterioles, veins, and venules) to the microscale (capillaries). Macroscale vasculature is generated on the temporal retina, starting from the central retinal artery (CRA) and ending in the central retinal vein (CRV). First, a statistical shape model^36^ of the major temporal arcades was developed using a fundus photographs dataset. The remaining superficial temporal vasculature was partially generated with a CCO algorithm.^26^

**Figure 1.**
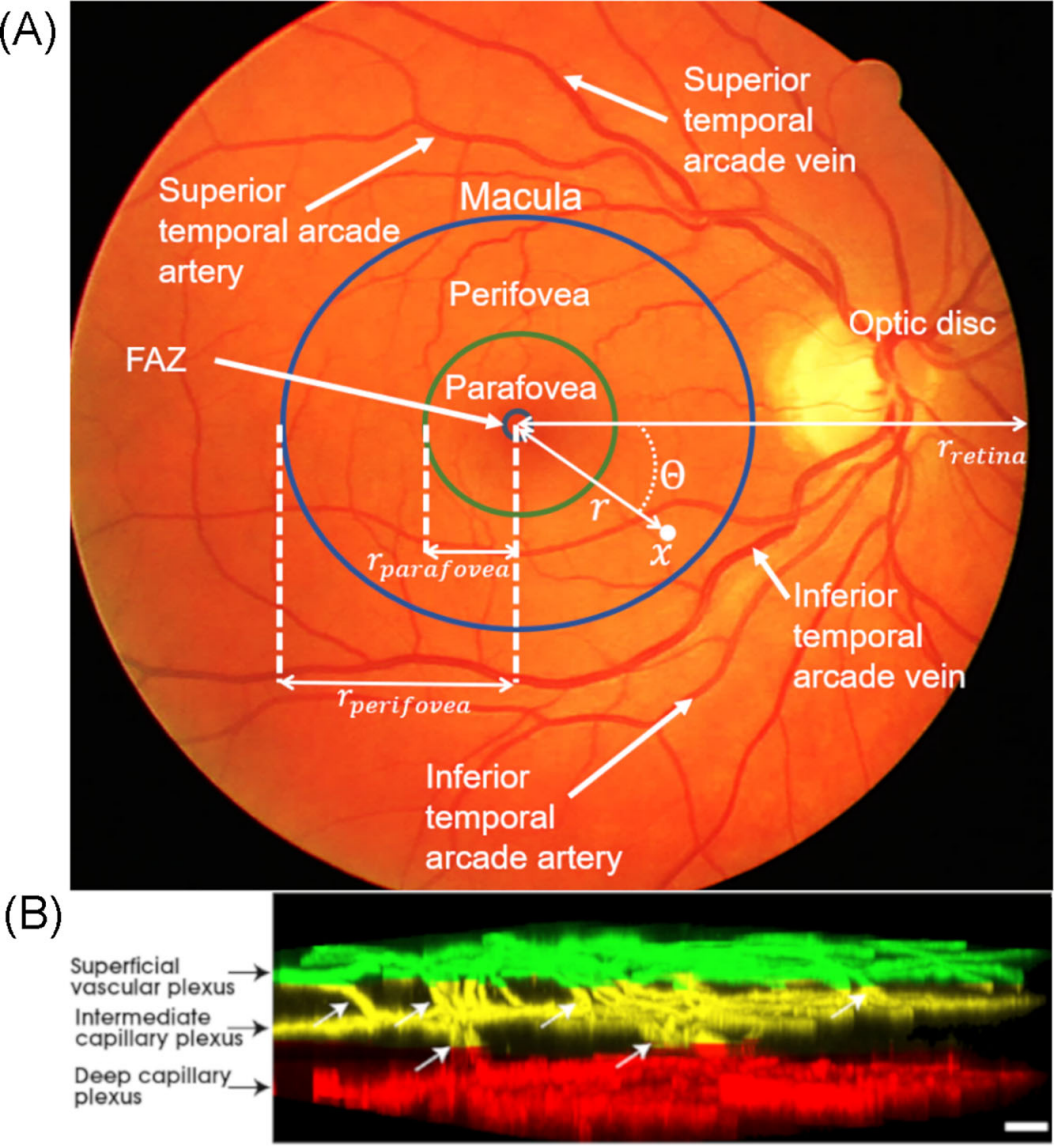
Architecture of the retina. (**A**) Landmarks of the retina on a 45° field-of-view colour fundus photograph from the DRIVE dataset.^34^ (**B**) Histology imaging of the three capillary layers of the macula. *White arrows* show connecting vessels between plexi. Image was adapted from the work of An et al.^35^ and is available under a CC BY-NC-ND license.

Microvasculature is generated in the macula area (see Fig. 1) across three vascular layers—namely, the superficial vascular plexus (SVP), intermediate capillary plexus (ICP), and deep capillary plexus (DCP), arranged as parallel, planar layers at fixed depths *z*. In contrast, the macrovasculature is only generated in the SVP. This is because the ICP and DCP are composed of capillaries in the perifovea and merge with the SVP outside the macula.^37^

#### Macrovasculature

##### Statistical Shape Model

Major temporal arcade vessels were manually segmented from the DRIVE dataset.^34^ The major temporal arcade vessels correspond to the four vessels (two veins, two arteries) that branch directly from the CRA and CRV and extend toward the superotemporal and inferotemporal quadrants of the retina, as shown in Figure 1A. Vessel centerline segmentations, from their branching at the level of the optic disc to the boundary of the fundus photograph (Fig. 1A, orange area), were extracted from eight color fundus photographs of eyes without retinopathy and centered at the fovea. Segmentation was performed using the Freehand Line tool in ImageJ 1.48 (National Institutes of Health, Bethesda, MD, USA).^38^ The pixel coordinates for each curve were extracted and translated so that the fovea was at the origin. This ensures that the model learns the distance between the optic disc and the fovea and between the arcades and the fovea, which may have importance in disease.^39^ For images of right eyes, curves are reflected across the *y*-axis so that all shapes correspond to left eyes (i.e., the optic disc will be on the left-hand side of the image). A simple principal component analysis–based statistical shape model learns the shape of all four temporal arcade vessels based on the location of inflexion points along the vessels (see Supplementary Material S1). The generated shapes are converted to length units using the rule of thumb for fundus photographs: 10° ≈ 5 mm, where the angle describes the field of view of the fundus camera. The generated vessels are linked to the CRV or CRA accordingly and assume initially the same radius as the central retinal vessels. The radius of the CRA is given in Table 1, and the radius of the CRV is larger by a factor of 1.1.1.^40^ The radii of vessels other than the CRA and CRV are updated during the generative process, as described below.

**Table 1.** List of Model Parameters and Their Ranges

Population Parameters	Description	Value (Mean ± SD)
*r_CRA_*	Radius of the central retinal artery (µm)	81 ± 8^49^
*v_CRA_*	Blood velocity in the central retinal artery (cm/s)	6.3 ± 1.2^49^
*MAP*	Mean arterial pressure (mmHg)	84 ± 6^49^
*IOP*	Intraocular pressure (mmHg)	11.1 ± 2.1^50^
**Macrovasculature Parameters**	**Description**	**Value (Stages 1, 2, 3)**

*N_terms_*	Number of terminal vessels, *n*	200, 150, 75
*p_pre-capillary_*	Target pressure at terminal vessels (mmHg)	30, 23, 23
*r_retina_*	Radius of the modelled region, centered around the fovea (mm)	15
*r_perifovea_*	Radius of the perifoveal (or macular region) (mm)	3
*r_parafovea_*	Radius of the parafoveal region (mm)	1.5
(σ, µ, *r*_0_)	Parameters of the log-normal distribution giving the distance of new terminal vessels from the center of the fovea as *r* = *r*_0_ + *e*^µ+σ^*^Z^*, with *Z* ∼ *N*(0,1)	4, –0.5, 0.02 for all stages
δ	Minimal radius symmetry ratio at bifurcations in Equation 1	0.8, 0, 0
θ*_min_*	Minimum bifurcation angle in Equation 1 (deg)	60, 60, 60
γ	Murray's law coefficient in Equation 1	3, 2.85, 2.85^22^^,^^41^
η	Starting point of the fixed-point iterations for the effective viscosity model used by the CCO algorithm^26^ (cP)	0.36^22^
*l_fr_*	*l_min_* correction step factor (Equation 2)	0.8, 0.5, 0.5
*v*	Perfusion area factor of the CCO algorithm (Equation 2)	1, 1, 1
*f_n_*	Neighborhood factor for finding candidate bifurcation points	1, 1, 1
**Microvasculature Hyperparameters**	**Description**	**Value (SVP, ICP, DCP)**

*N_seeds_*	Number of seeds used to generate a capillary bed, *n*	5500, 16,000, 10,500
*r_capillary_*	Radius of capillaries (µm)	2.5, 2.5, 2.5
*r_FAZ_*	Radius of the foveal avascular zone (mm)	0.25, 0.25, 0.25^51^
*z*	Plexus depth (µm)	0, 135, 180^51^

##### Arterial/Venous Branching Trees

The statistical shape model produces pairs of temporal arcades: one arterial linked to the CRA, one venous linked to the CRV. This section describes how an arterial and a venous tree is generated for each arcade. At this stage, trees are structured branching trees using a space-filling algorithm. The algorithm is a modification of the CCO algorithm.^26^ With the CCO, trees are grown to minimize the total volume of the tree while, for each addition of vessel segments, keeping a constant pressure drop from inlet to outlet and satisfying several geometrical constraints. The hemodynamic model used by the CCO algorithm is similar to Equations 5 to 7 but with a different viscosity model.^26^ Viscosity is computed with a fixed-point scheme with starting point η. The geometrical constraints affect the radius of branches,^41^ the symmetry of branches, and the branching angle and aspect ratio of new segments.^26^ Specifically, when *r_p_*, *r*_1_, and *r*_2_ represent the radius of the parent vessel and the two daughter branches, respectively, and θ is the angle between the two branches, the following constraints apply:

(1) $$\begin{array}{c} r_{p}^{\gamma }=r_{1}^{\gamma }+r_{2}^{\gamma },\;\frac{\min\left(r_{1},r_{2}\right)}{\max\left(r_{1},\;r_{2}\right)}>\delta \;\;\;\mathrm{and}\;\;\;\theta >\theta_{min}. \end{array}$$

Further constraints ensure that the generated tree provides a relatively homogeneous coverage of the domain. Namely, for a tree grown in a domain Ω ⊂ R^2^ with *N* terminal vessels, the location for a new terminal vessel is rejected if it is less than

(2) $$\begin{array}{c} l_{min}={\left(\frac{\nu }{\pi \left(N+1\right)}\int_{\Omega }dA\right)}^{1/2}. \end{array}$$

If 20 consecutive points fail to satisfy Equation 2, *l_min_* is reduced by a factor *l_fr_*.

In addition to the original CCO algorithm constraints, tree growth is geometrically constrained to prevent vessels from crossing the line that passes through the optic disc center and the center of the fovea, which separates the superior and inferior halves of the retina. Additionally, we used a custom log-normal probability distribution (see Table 1) to select the location of new vessel segments to mimic an angiogenic process biased toward the fovea while keeping the foveal avascular zone (FAZ) free of vessels. Indeed, the fovea has a higher concentration of cells and therefore greater metabolic needs compared to the rest of the retina.^42^ For a candidate terminal vessel located at *x_new_*, the CCO algorithm finds the most suitable vessel within a radius $f_{n}\times {\left(\int_{\Omega }dA\right)}^{1/2}$ of *x_new_* that satisfies the above geometrical constraints.

The CCO algorithm is applied in three stages, within three circular regions: in a disk of radius *r_retina_* (i.e., the entire computational domain), in an annulus with radii *r_parafovea_* and *r_perifovea_*, and finally in a disk of radius *r_parafovea_* (Fig. 1).

To simulate growth biased toward the fovea while keeping the FAZ free of vessels, the coordinates of a segment endpoint are given by *x* = (*r*cosθ, *r*sinθ), where θ is the opening angle and follows a uniform distribution over the interval [0, 2π] and *r* is the distance to the center of the macula, as shown in Figure 1A, and follows a log-normal distribution (see Table 1). Arteriovenous networks of the superficial vascular plexus are generated in three steps: (1) the CCO algorithm is applied to create a backbone of larger arterioles and venules from the arterial and venous arcades. The arterial and venous backbones are grown separately in the first step. For each tree, the CCO algorithm requires volumetric blood flow at the root (CRA or CRV) and a pressure drop across the vasculature. Blood flow in the CRA is computed from its radius and blood flow velocity. From conservation of mass, blood flow is the same in the CRV. Ocular perfusion pressure (*OPP*) refers to the pressure drop between the CRA and CRV, namely:

(3) $$\begin{array}{c} OPP=p_{CRA}-p_{CRV}. \end{array}$$

Pressure in the CRV is assumed equal to intraocular pressure (*IOP*).^21^^,^^43^^,^^44^ Pressure in the CRA is estimated from the mean arterial pressure (*MAP*)^21^^,^^43^^,^^44^:

(4) $$\begin{array}{c} p_{CRA}=\frac{2}{3}MAP. \end{array}$$

The pressure drops across the vascular trees are set to *p_pre-capillary_* – *p_CRV_* for the venous tree and *p_CRA_* – *p_pre-capillary_* for the arterial trees. The value of *p_pre-capillary_* is taken from pressure in pre- and postcapillary vessels in the theoretical model by Takahashi et al.^22^

#### Microvasculature

At the macroscale, arterioles and venules generally follow a bifurcating structure, with parent vessels giving rise to two daughter branches. The CCO algorithm follows this logic to create vascular trees. At the microscale, however, capillaries tend to form complex nets, forming loops and anastomoses^35^ that are incompatible with the logic of the CCO algorithm. Therefore, we adopted the method proposed by Linninger et al.^25^ to generate capillary beds connecting the arterial and venous trees. In short, a disk of the size of the macula (see Table 1) is meshed with a Delaunay triangulation generated from *N_seeds_* randomly sampled points within the disk. The centroids of the triangles are used to generate a Voronoi diagram. In brief, a Voronoi diagram partitions the plane into polygonal regions centered around input points. The edges of the polygons form the capillary bed. In the SVP, capillaries coexist with arterioles and venules but should not intersect them; therefore, capillaries intersecting with arterioles or venules are removed from the capillary bed. This also creates a capillary-free region that is found surrounding arterioles in the SVP.^35^

In the SVP, a proportion α of arterioles and venules and all terminal vessels within the macula are connected to the nearest capillary. Because of the lack of specific data, α was arbitrarily set to 40% in all simulations unless specified otherwise.

Interplexi connections are subject to debate.^18^^,^^35^^,^^45^ Because the ICP and DCP are modeled in the macula only, interplexi connections are based on the findings by An et al.^35^ in the parafovea. Specifically, arterioles and venules within the macular area of the SVP bifurcate to the ICP, and those branches immediately bifurcate to the DCP. This corresponds to the most prevalent patterns in the histology study.^35^ From the SVP, 30% of the arterioles and venules were selected for bifurcation to the ICP, and the bifurcation points were added in the middle of the selected vessels.

All capillary segments are initially given the same radius (*r_capillary_*) unless they are connected to an arteriole or venule, in which case their initial radius is twice that of other capillaries. Diameter transitions at bifurcations are smoothed using the method proposed by Linninger et al.^25^ In short, the diameter of a segment becomes the average of the diameters of itself and of the parent and daughter branches.

When all vascular segments have been assigned a radius, the last necessary step for hemodynamics simulation is to find the flow direction in the capillary network created by the Voronoi diagram. This can be achieved by using the diffusion equation. Representing the vasculature as a graph, values are assigned to nodes: 1 for arterial nodes, 0 for venous nodes, and 0.5 for capillary nodes. The graph Laplacian of the vasculature is used to update the nodal values, creating gradients along edges (vessel segments). These gradients provide an ordering of the capillaries, from high to low value, which ensures that the graph stays acyclic, which is a necessary condition for hemodynamics simulations.

### Hemodynamics Model

**Figure 2.**
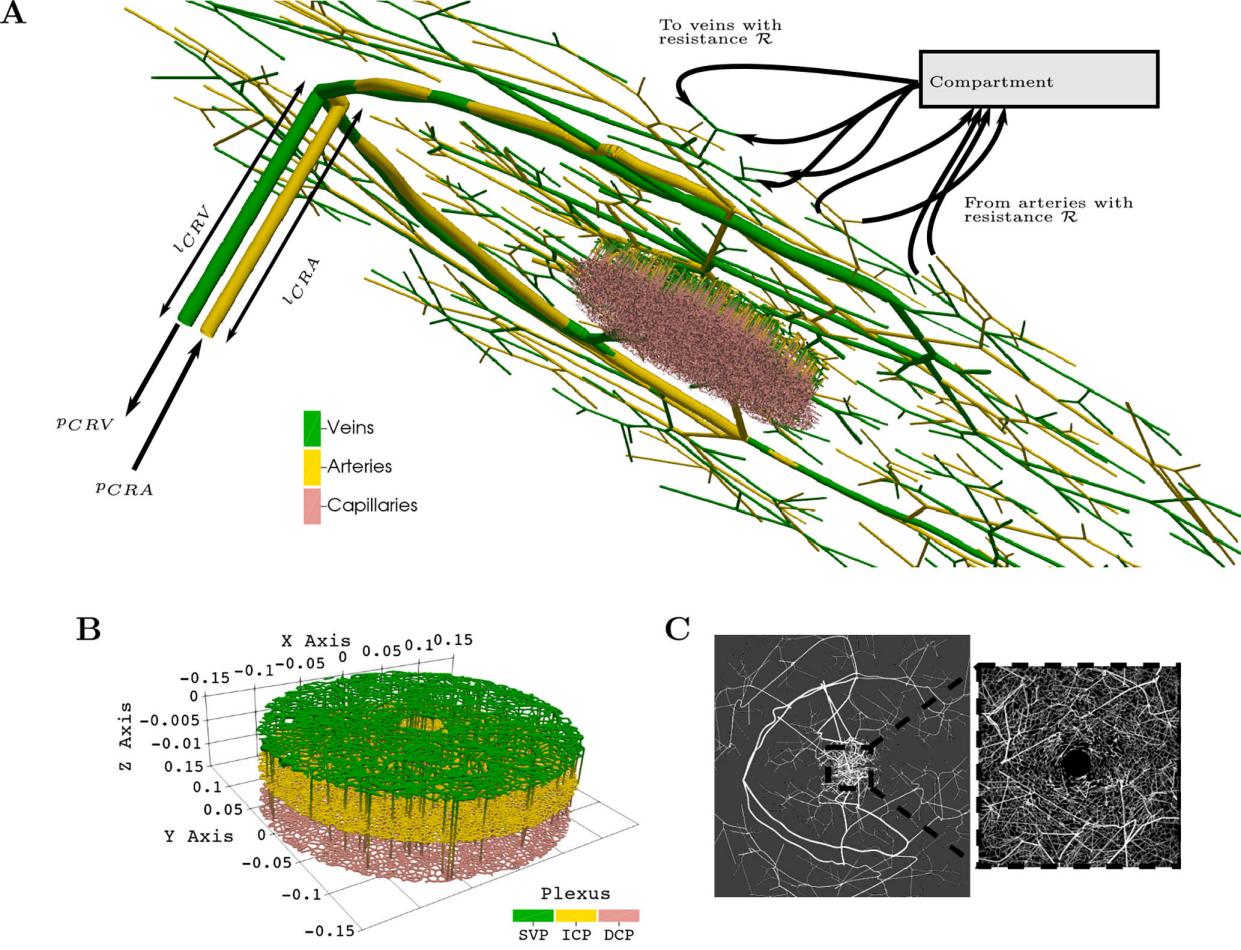
(**A**) A visualization of one generated vasculature. The hemodynamics simulations use blood pressure at the CRA and CRV as inputs. Outside the macula, terminal arteries are linked to a compartment through an artificial resistance R which redistributes the flow to terminal veins through connections with the same resistance R.. In the macula, capillaries connect arteries to veins across three vascular plexi. (**B**) Zoomed-in view of the dense macular region from **A**. (**C**) En face view of the generated SVP in the temporal region and the macula (zoomed-in inset).

Blood is modeled as an incompressible, Newtonian fluid flowing in a network of connected tubes by the Hagen–Poiseuille equation. This modeling framework considers the vasculature as an arrangement of connected, straight tubes (see the example shown in Fig. 2A), across which pressure drop ∆*p*, vascular resistance *R*, and volumetric blood flow *Q* are related by

(5) $$\begin{array}{c} Q=\frac{\Delta p}{R}. \end{array}$$

Vascular resistance is a function of tube radius *r*, length *l*, and blood viscosity µ, as follows:

(6) $$\begin{array}{c} R=\frac{8\mu l}{\pi r^{4}}. \end{array}$$

Blood is a non-Newtonian fluid and is subject to the Fåhræus–Lindqvist effect^46^ in the microcirculation. Non-Newtonian effects are accounted for by a diameter- and hematocrit-dependent, empirical, effective viscosity law^47^:

(7) $$\begin{array}{c} \mu \;\left(D\right)=\left[1+\left(\mu_{0.45}-1\right)\frac{{\left(1-H_{D}\right)}^{C}-1}{{\left(1-0.45\right)}^{C}-1}{\left(\frac{D}{D-1.1}\right)}^{2}\right]{\left(\frac{D}{D-1.1}\right)}^{2},\; \end{array}$$

where *D* is the vessel diameter in microns and *H_D_* is the discharge hematocrit:

(8) $$\begin{array}{c} \mu_{0.45}=6e^{-0.085D}+3.2-2.44e^{-0.06D^{0.645}}, \end{array}$$

and

(9) $$\begin{array}{ccc} & & \;C=\left(0.8+e^{-0.075D}\right)\left(-1+{\left(1+10^{-11}D^{12}\right)}^{-1}\right)\\ & & \;+\;{\left(1+10^{-11}D^{12}\right)}^{-1}, \end{array}$$

the discharge hematocrit is kept constant at 45% in this work. Considering the relatively small caliber of retinal vessels, Equations 5 to 9 apply to the entire vasculature.^47^

In the macular area, the vasculature is fully connected, with arterioles connecting to venules through capillaries; therefore, no boundary conditions are needed. Outside the macula, however, the first stage of the CCO algorithm detailed earlier leaves terminal vessels with no further branches on the arterial and venous sides. This happens as a result of not generating the full extent of the peripheral vasculature. To close the vascular network, these terminal vessels must somehow be linked to the CRA/CRV, or hemodynamics in those vessels need to be explicitly given as boundary conditions of the model. In the absence of adequate data on hemodynamics for those vessels, we chose to instead link the terminal vessels through a compartment, as shown in Figure 2A. This avoids the need for boundary conditions. All terminal arteries outflow into the compartment through artificial vessels with a resistance *R*. All terminal veins drain the vascular compartment through artificial vessels with the same resistance. For baseline simulations, *R* = 1 × 10^6^ mmHg s*/*mL was selected such that the hemodynamic parameters’ distribution across the vasculature is similar to experimental data.^19^^,^^48^

### Validation Metrics

Six morphological metrics were used for comparison with OCTA:

• Four of the indices proposed by Chu et al.^2^: vessel area density (VAD), vessel skeleton density (VSD), vessel diameter index (VDI), and vessel complexity index (VCI), computed according to Equation 10
• Fractal dimension (FD), computed with a box-counting method^4^
• Intervessel distance (IVD), computed with Euclidean distance transform.^52^

Only VAD and FD were used in the ICP and DCP.

For each plexus, VAD was used in the model development stage to determine appropriate values for two of the hyperparameters: *N_terms_* and *N_seeds_* (see Table 1). Both IVD and FD require skeletonized, pixelized images of the vasculature to be computed. To create those, generated macular vessels are mapped to a white canvas, then saved as binary images.

The Euclidean distance transform was used to compute IVD, and a box counting method was used to estimate FD. The remaining metrics were computed from the cumulated length ($L=\sum_{i\in V}l_{i})$ and cross-section area of vessels $\left(A=\sum_{i\in V}2r_{i}l_{i}\right)$ within a given plexus and inside a field of view of area *X* as follows:

(10) $$\begin{array}{c} VAD=\frac{A}{X},\;VSD=\;\frac{L}{X},\;VDI=\frac{A}{L},\;VCI=\frac{{\left(2L\right)}^{2}}{4\pi A}. \end{array}$$

These metrics have been proposed as proxies for the average caliber, length, and overall complexity and quality of the vasculature on an OCTA.^2^

Vessels are assigned a stream order, or Horton–Strahler order. In brief, capillaries are assigned an order of 0, and then, moving upstream for arteries or downstream for veins, the orders are assigned as follows^35^:

• If the vessel has one branch of order *i* and all other branches are of order less than *i*, then the order of the vessel is *i*
• If the vessel has two or more branches of order *i* and *i* is the largest order among the branches, then the order of the vessel is *i* + 1.

From the hemodynamics simulations, two variables were extracted to quantify macular perfusion: *retinal blood flow*, defined as the volumetric flow rate of blood entering the retina, and the *macular flow fraction*, defined as the percentage of retinal blood flow entering the macula. Spearman correlation coefficients were calculated for both hemodynamics variables and against each morphological metric. We derived 95% confidence intervals (CIs) using bootstrapping (*N* = 1000).

### Sensitivity Analysis

The method presented in this work relies on several hyperparameters. Those parameters are described below and in Table 1 and in more detail in Talou et al.^26^ for CCO algorithm–specific parameters. The values for these parameters either are unknown (e.g., *N_terms_*) or are subject to uncertainty in their measurement (e.g., δ, γ). We performed a variance-based sensitivity analysis to decompose the variance in the output of the model (*Var*[*Y*]). Sobol indices summarize the importance of sets of inputs *X_i_* with indices between 0 and 1.^53^ In this work, we report first (*S_i_*) and total (*S_T__i_*) order indices, which are often enough to understand parameter importance.^53^ In short, *S_i_* quantifies the contribution of *X_i_* alone, and the *S_T__i_* quantify its total contribution—namely, its first-order contribution plus all the higher order contributions.^53^ Further details can be found in the Supplementary Material.

### Uncertainty Quantification

Uncertainty quantification aims at assessing the credibility of the prediction of a model.^54^^,^^55^ For the hemodynamics model, uncertainty stems from two parameters: *OPP* (Equation 3) and *R*. To quantify the uncertainty brought on by the correlation coefficients, the same experiment was reproduced for 45 different scenarios where

• The resistance parameter *R* was set to 5 × 10^5^ mmHg s*/*mL, 1 × 10^6^ mmHg s*/*mL, and 5 × 10^6^ mmHg s*/*mL.
• *OPP* was set to 80%, 100%, and 120% of its baseline value
• The fraction α was varied between 0*.*2 and 0*.*6 by increments of 0*.*1.

## Results

A virtual population of 200 healthy vasculatures was generated. The population parameters were sampled from normal distributions as specified in the “Population Parameters” section of Table 1. All other parameters were kept at their baseline values, given in Table 1. Hemodynamics simulations were performed for each virtual vasculature using the virtual individual's *OPP*. The mean ± SD for *OPP* was 45.2 ± 4.2 mmHg (range, 32.83–56.0). The parameter *v_CRA_* was not used in the hemodynamics simulations.

### Validation of the Network Structure and Hemodynamics

**Figure 3.**
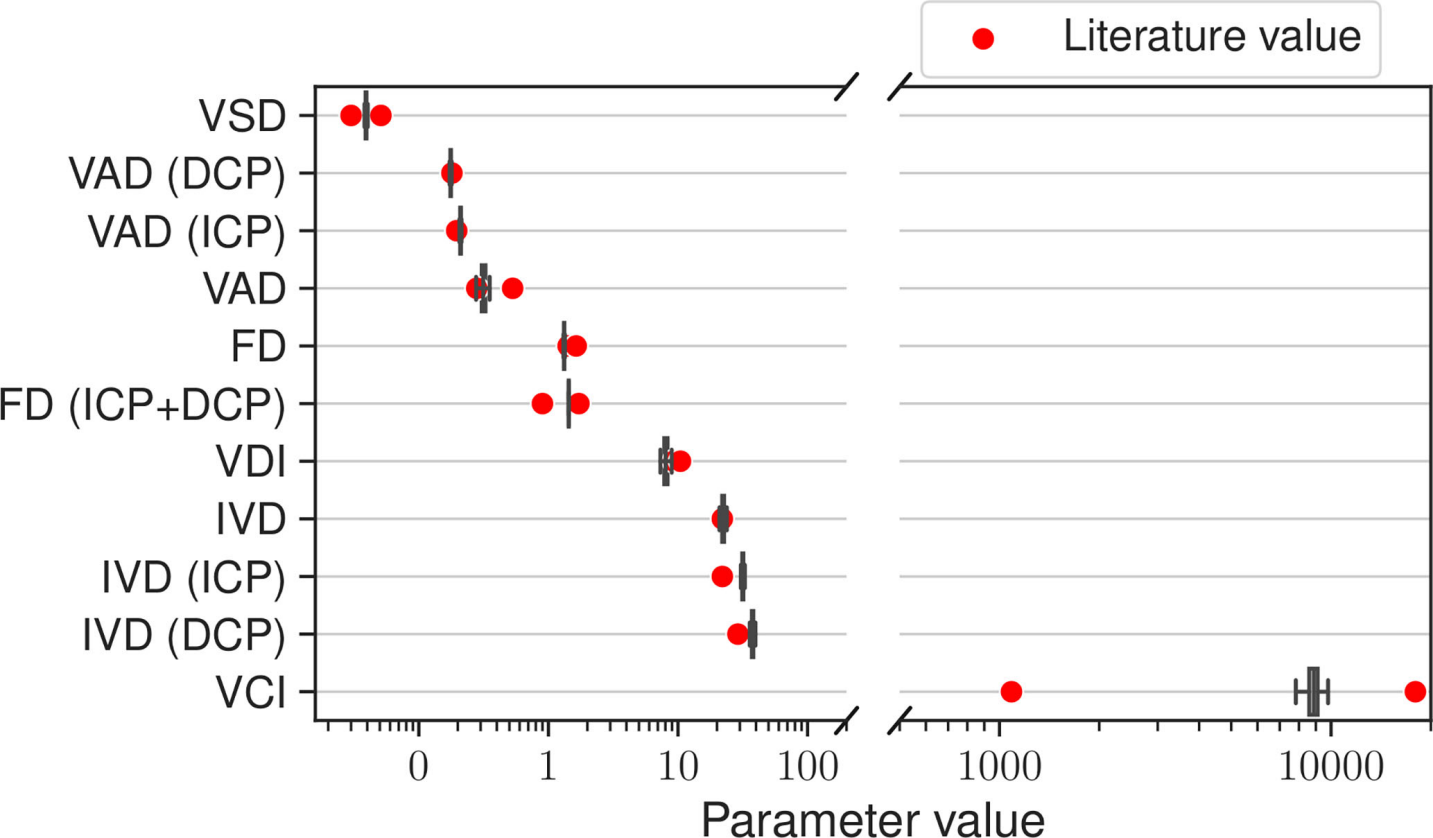
Comparison of the morphology of virtual vasculatures in the macula against OCTA measurements. *Dots* show the mean value reported by independent studies (VAD^56^, VSD^56^, VDI^57^, IVD^52^, FD4^58^ in the SVP; VAD^59^, FD4^58^ for the ICP and DCP) for healthy eyes. Model values are shown as box and whisker plots. Whiskers extend to the minimum and maximum within 1.5 times the interquartile range. Unless otherwise specified, the metrics relate to the SVP.

The morphology of the macula, within a disk of diameter 3 mm centered at the fovea, is compared to literature values of the same parameters computed on OCTAs^2^^,^^4^^,^^52^ in Figure 3.

**Figure 4.**
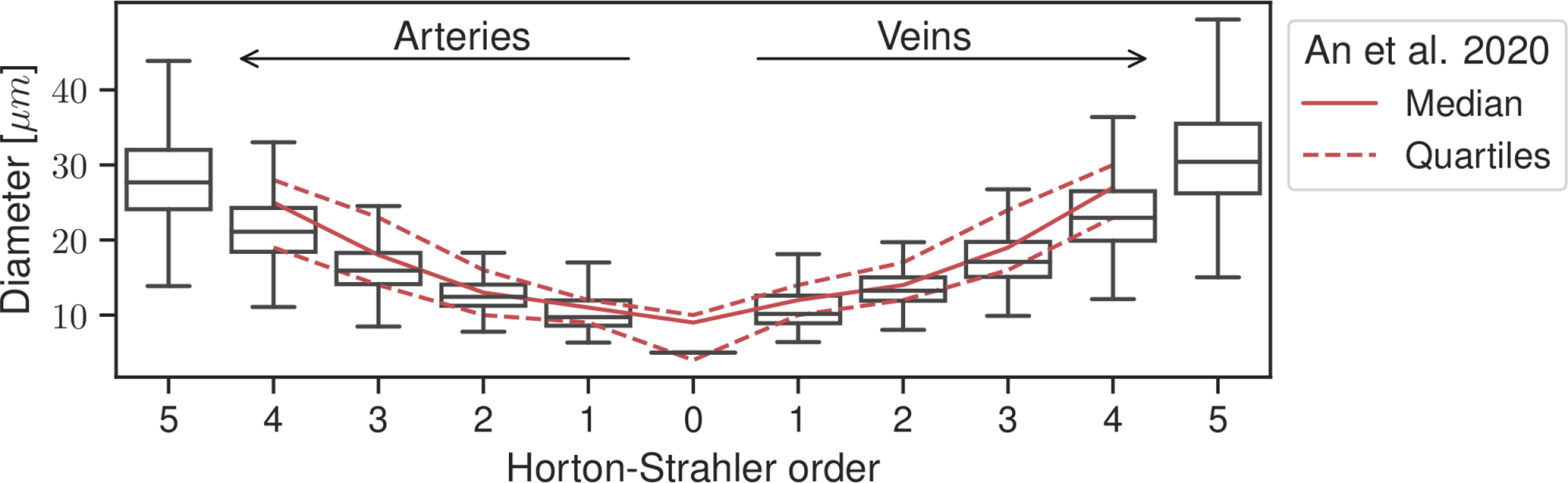
Box-and-whisker plot of the diameter of virtual vessels in the macula for each Horton–Strahler order. On average, close to 5000 vessel segments were analyzed for each vasculature. Whiskers extend to the minimum and maximum within 1.5 times the interquartile range. For comparison, lines show the mean (*solid line*) and quartiles (*dashed lines*) from experimental data.^35^

Figure 4 shows vessel diameters for each stream order in the macula. The distribution is similar to histological data.^35^ Mean diameters were smaller in the model but lay within the reported ranges for each order. The ratio of average arteriole diameter to average venule diameter increased from 0.92 ± 0.09 in order 5 vessels to 0.95 ± 0.05 in order 1 vessels. For all orders combined, the ratio was 0.937 ± 0.031, which is consistent with experimental measurements of 0.9 ± 0.1.^40^

**Figure 5.**
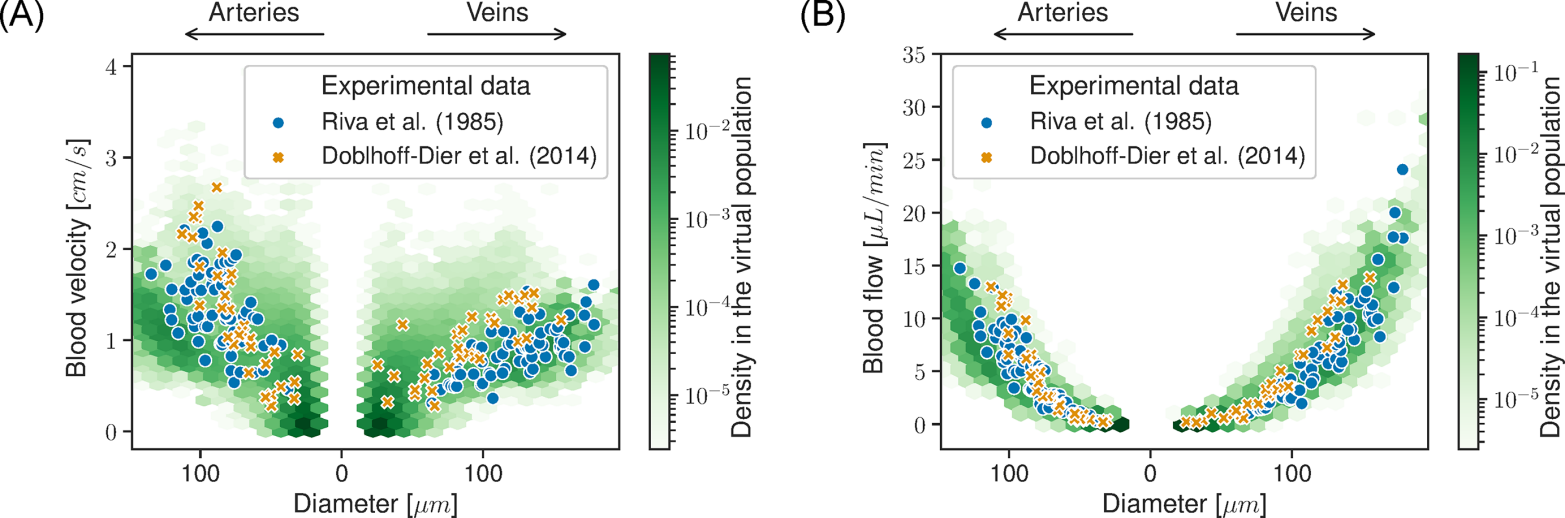
Blood velocity (**A**) and volumetric blood flow rate (**B**) distributions against vessel diameter in the virtual vasculatures compared with two independent experimental studies.^19^^,^^48^ Blood vessels from the simulations are binned into *hexagons*. *Color* *maps* show the probability density across the 200 virtual individuals.

From the hemodynamics simulations, the means ± SD for retinal blood flow, blood velocity in the CRA, and macular flow fraction were 20.80 ± 7.86 µL/min (range, 5.01–60.45), 1.62 ± 0.30 cm/s (range, 0.87–2.6), and 15.04% ± 5.42% (range, 4.92–32.74). On average, retinal blood flow in the model was lower compared to experimental studies that have reported means of 30 to 40 µL/min.^19^^,^^48^ Blood velocity in the CRA was also lower in the model compared to the average of 6.3 cm*/*s reported by experimental work.^49^ Figure 5A compares blood velocity along the vasculature with experimental studies.^19^^,^^48^ Additionally, these studies reported volumetric blood flow rates against diameter. These distributions are compared with those of the model in Figure 5B. In the venous circulation, model predictions of flow and velocity were consistent with experimental data. On the arterial side, both velocity and flow were visually lower compared to the same studies.

### Four Structural Variables Are Strongly Linked to Retinal Function

**Figure 6.**
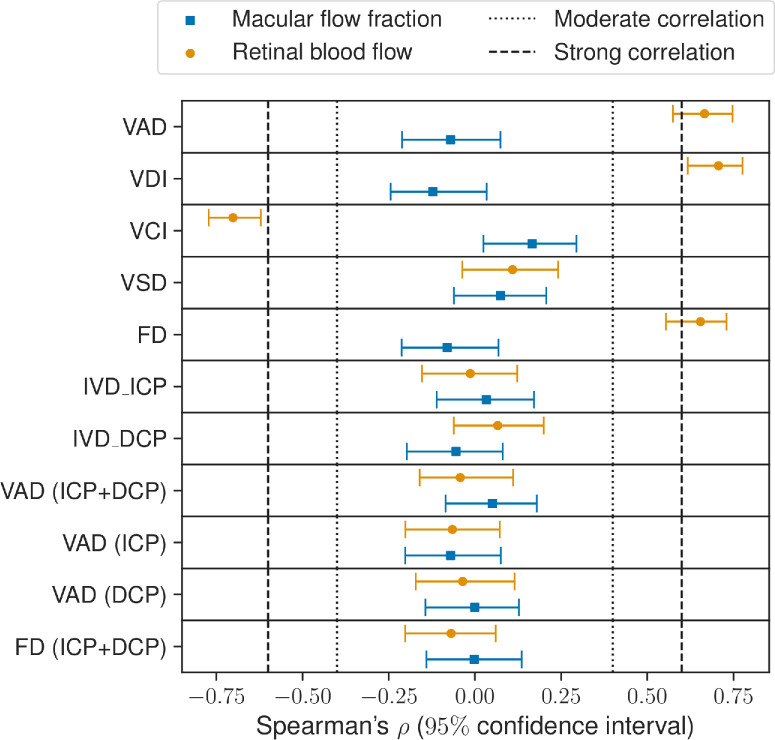
Spearman correlation coefficients testing for monotonous correlations between morphology and hemodynamics of the macular vessels. Values closer to 1 or –1 indicate stronger correlations. The 95% CIs were estimated using bootstrapping.

We next look to understand how the morphology of the macular vasculature affects the hemodynamics of the retina and of the macula using our model. Figure 6 shows the Spearman correlation coefficients for each variable. Vertical lines show the threshold typically used for a correlation to be considered moderate (dotted lines) and strong (dashed lines). For the healthy virtual cohort, the model found only VAD, VDI, VCI, and FD of the SVP to be predictors of retinal blood flow. The correlations with the macular flow fraction were all below 0.25, showing weak or no correlations.

### Sensitivity Analysis and Uncertainty Quantification

#### Minimum Branching Angle Dominates Structural Variability

**Figure 7.**
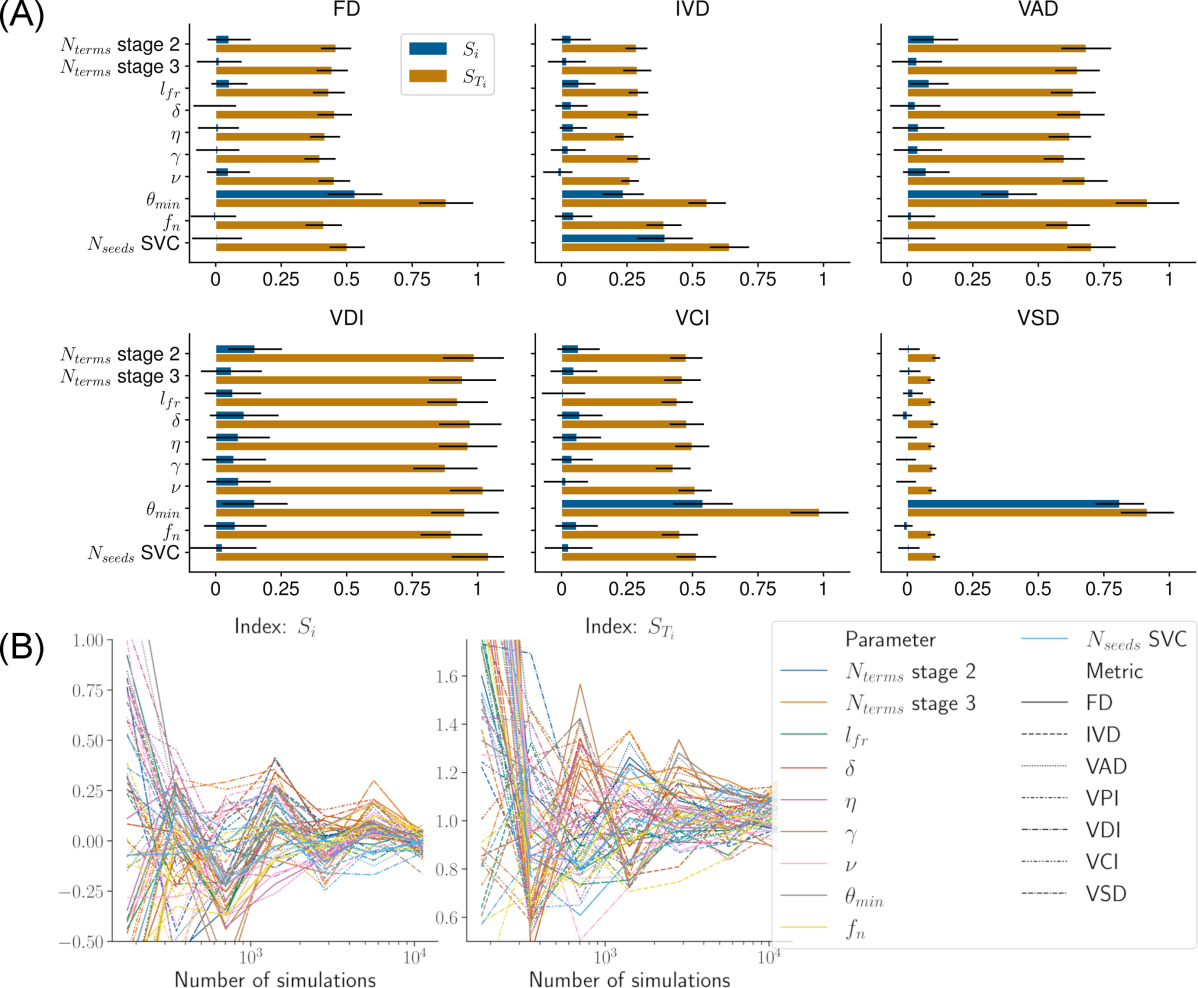
Results of the global sensitivity analysis. (**A**) The *bars* show the first-order (*S_i_*) and total-order (*S_Ti_*) Sobol indices for each hyperparameter of the method with respect to each morphological metric of the superficial vascular plexus. The *error bars* show the 95% CIs for the indices. (**B**) Convergence of the indices was checked by plotting the values of the indices with an increasing number of simulations used for their computation.

 Figure 7A shows the Sobol indices for 10 hyperparameters computed with the Python library SALib^60^^,^^61^ using around 11,000 simulations. The parameters were sampled uniformly within the ranges presented in Table 2 using the algorithm developed by Saltelli.^62^

**Table 2.** Ranges for the Hyperparameters for the Computation of Sobol Indices

*N_terms_* Stage 2	*N_terms_* Stage 3	*l_fr_*	**δ**	**η**	**γ**	*v*	θ*_min_*	*f_n_*	*N_seeds_* SVP
[300, 500]	[200, 400]	[0.1, 0.9]	[0.1, 0.9]	[0.2, 0.5]	[0.2, 0.5]	[2, 3]	[0, 72]	[0, 5]	[300, 700]

For most metrics, all parameters share a similar total order and small first order. Notably, θ*_min_* stands out as the most influential overall, explaining around 50% of the variance in FD and most of the variance in VSD (*S_i_* = *S_Ti_* ≈ 1) by itself. Other parameters of interest include *N_seeds_* for the SVP, *N_terms_* or the second stage of the CCO algorithm, and, to a lesser extent, *l_fr_*. These results indicate that those four parameters are enough to produce a virtual population with interpopulation variability, at least in the SVP.

#### Macular Flow Fraction Is Independent of OPP

The results presented earlier rely on several hypotheses and parameters that introduce a degree of uncertainty. Across all scenarios, the mean total retinal blood flow varied between –30.0% and 38.3% of baseline values, and the macular flow fraction varied between –47.1% and 12.9%. The results for the nine scenarios where α was kept at its baseline value are shown in Supplementary Table S1. Variation in *OPP* had almost no effects on macular flow fraction (Pearson's *r*^2^ < 10^–3^ for all values of *R*) but was linearly correlated with total retinal blood flow (*r*^2^ > 0.89 for all values of *R*). The coefficients are given for those nine scenarios in Supplementary Table S2. Parameter α was not correlated with either of the hemodynamic variables (*r*^2^ < 10^–2^ for all variations of *OPP* and *R*).

## Discussion

A virtual cohort of 200 healthy patients was generated and analyzed. The generated vasculature showed good agreement, structurally, with experimental and clinical measurements. The hemodynamics model showed good qualitative and quantitative agreement with two independent experimental studies. The model predicted strong associations between several microvascular parameters and total retinal blood flow.

### Validation

We validated the models against experimental data. The morphology of the SVP was within the ranges of values found in the literature for healthy retinas, as quantified by OCTA.^2^^,^^4^^,^^52^^,^^56^^,^^58^ In the ICP and DCP, VAD was very close to values reported in a histology study,^59^ but IVD was larger in both plexuses compared to OCTA data.^52^ This was more marked in the ICP compared to the DCP. The morphology of the microcirculation delineated on OCTA is sensitive to several factors, including scan postprocessing^56^^,^^57^ and segmentation of the different plexuses, which makes direct comparison complicated.

The morphology of the ICP and DCP vasculature was very homogeneous across the generated cohort, as indicated by the small standard deviations in Figure 3. As demonstrated in the SVC by the sensitivity analysis, this may be resolved by varying *N_seeds_*, although reasonable bounds need to be defined.

Figure 4 shows that the distribution by the model of diameters across Horton–Strahler order was similar to a histological study.^35^ However, capillaries were smaller in our model compared to the data. In the study by An et al.,^35^ capillaries were any vessel with diameter smaller than 8 µm. In our model, capillaries were assigned a diameter of 5 µm or 10 µm if they were directly connected to an arteriole or venule. This strategy may be too simplistic to represent the spread of diameters in the vascular bed. Others have suggested updating the diameter of vessels based on blood pressure from a first hemodynamics simulation.^25^ More in-depth analysis of the capillary beds is necessary in order to develop an appropriate strategy. In the meantime, sensitivity analysis and uncertainty quantification can help improve the reliability of the model.

Figure 5 shows that the predictions by the model of blood velocity and flow across the vasculature were consistent with experimental data. However, for the arterial circulation, both flow and velocity were slightly lower in the model.^19^^,^^48^ Similarly, blood flow and velocity in the CRA were both lower in the model compared to experimental data.^19^^,^^48^^,^^49^^,^^63^ As seen in Equations 5 and 6, blood flow and velocity are respectively proportional to the fourth and second power of vessel radius. Therefore, an increase in radius by a factor of $\sqrt{2}$ ≈ 1.4 for velocity and $\sqrt[{4}]{2}$ ≈ 1.18 for flow would be sufficient to double the predictions of the model. It is unclear whether vessel diameter measurement in experimental studies^19^^,^^48^ has included the vessel wall in the measurement. We assumed that the diameters were those of vessel lumen, which could lead to an overestimate of lumen radii between 20% and 35% for larger temporal arteries.^64^^–^^66^ In experimental studies, the same relation between flow and radius is assumed, and blood flow is estimated from velocity *v* and diameter *D* measurements as *Q* = *v*π*D*^2^/4. Therefore, even a small measurement error in vessel diameter combined with error in measurement in velocity still results in large deviations from the true blood flow. Both measurements are challenging and prone to errors.^67^ To test this hypothesis, we reduced by 20% the lumen diameter of arteries larger than 100 µm in diameter and ran the hemodynamics simulations for the entire population. The velocity and flow distributions for this experiment are provided in Supplementary Figure S1 and show improved agreement with the experimental data. In addition, all parameters in our virtual populations were sampled from independent normal distributions, which is likely an incorrect assumption as, for example, vessel diameter is likely to be correlated with arterial pressure and *IOP*.^68^ As discussed by Doblhoff-Dier et al.,^19^ studies have reported average total retinal blood flow ranging from 30 to 80 µL/min.^19^^,^^48^^,^^63^ Despite the uncertainty in measurements, most studies seem to agree on values in healthy eyes of around 30 to 40 µL/min.^19^^,^^48^ Despite the difference in hemodynamics in the CRA, Figure 5 shows that the discrepancy with experimental data is reduced as the vessels branch out. Similar to the study by Doblhoff-Dier et al.,^19^ blood velocity seems to scale linearly with diameter for larger vessels, but this trend is lost in smaller vessels. The overall lower blood velocity can be attributed, as discussed above, to the discrepancy in velocity in the CRA.

The simulations found that macular flow fraction was between 2.51% and 11.54%; however, to best of our knowledge, there are no data to validate these values. The macula has twice the density of cells compared to the rest of the retina.^42^ Assuming that regions of higher cell density require similarly higher blood flow, it can be estimated that the macula requires 15% to 30% of the total retinal blood flow, although these are rough estimates.

We quantified the effects of the two parameters of the hemodynamics model on total retinal blood flow and macular flow fraction. Increasing *R* is similar to gradually closing the connections to/from the vascular compartment; therefore, flow is shunted toward the macula, and macular flow fraction increases. Decreasing *R* has an opposite effect. However, the macular flow fraction will eventually reach a plateau when the CRA reaches its maximum capacity in terms of blood flow: Regardless of the resistance of paths outside the macula, blood will flow through the macula, and total retinal blood flow is bounded by physical constraints. Indeed, in our model, for a given *OPP*, flow in the CRA is theoretically bounded by the radius and length of the CRA according to Equation 5. The same effect explains the non-symmetrical changes in total retinal blood flow as *R* is decreased (see Supplementary Fig. S2).

### Associations Between Vascular Structure and Function

Analysis of the 200 virtual vasculatures revealed associations between several of the morphological metrics and total retinal blood flow. Larger VAD, VDI, and FD in the SVP were strongly associated with larger retinal blood flow. In contrast, larger VCI in the SVP was strongly correlated with smaller retinal blood flow. Interestingly, VAD in the ICP showed a moderate positive correlation with retinal blood flow. However, VAD in the DCP and in the combined ICP–DCP complex, as well as FD in the ICP–DCP complex, did not show any significant correlations. None of the tested metrics was significantly associated with the fraction of flow transiting through the macula. Uncertainty quantification showed that those results were independent of the parameters of the hemodynamics model. As shown in Supplementary Figures S3 and S4, varying *OPP*, *R*, and α does not have any effect on the Spearman correlation coefficients.

The correlation coefficients between morphological metrics and hemodynamics were obtained from one-to-one comparisons and therefore do not capture possible interplay between morphological metrics. Additional analysis is required to better understand which metrics or combination of metrics are strong predictors of blood flow.

### Developing VPs With a Smaller Parameter Space

We have presented the results of a global sensitivity analysis of the hyperparameters of our method on the morphology of the vasculature in the SVP. The results are presented as first-order and total-order Sobol indices for 10 inputs and six outputs in Figure 7A. These indices were extracted from a large number of simulations in order to ensure convergence, which was reached with around 8000 simulations, as seen in Figure 7B.

The total-order indices were globally similar for all parameters and added little explanation to the importance of parameters. Computing second-order indices may be necessary to reduce the number of parameters before attempting to generate a different population or to pursue uncertainty quantification. However, second-order indices require a larger number of simulations to achieve convergence with Monte Carlo methods and are therefore expensive to compute.^53^ In this case, developing a surrogate model (e.g., polynomial chaos expansion) might be required.^53^ Nonetheless, the first-order and total-order indices suggest that the parameter space can be reduced to as little as four parameters, depending on which morphological metric is deemed more important.

Interestingly, θ*_min_* appeared to be the most influential parameter. In particular, it was the sole parameter influencing VSD. This result supports the hypothesis by Xao et al.^30^ that branch geometry is correlated with the vessel perimeter index, which would be twice VSD when computed on artificial vessels. The associations between vascular structure and hemodynamics should be investigated further, perhaps with spatial metrics that can be compared with OCTA measurements.^30^

### Limitations

Our method has several limitations that should be acknowledged. We did not model the peripapillary capillary plexus, ignored the curvature of the retina, and assumed a unique interplexus connection pattern. We also assumed that each plexus lay in a two-dimensional plane. Additionally, direct connections between arterioles/venules and capillaries in the SVP were added with a likelihood α, which ignores physiological behaviors. Similarly, the likelihood of an arteriole or venule of bifurcating to the deeper layers was also arbitrarily set to 30%. The validity of these two assumptions has yet to be evaluated.

The effects of uncertainty in measurements (i.e., *MAP*, *IOP*, *r_CRA_*, and *v_CRA_*) on the generated vasculature remain to be quantified. The number of hyperparameters is large, and global sensitivity analysis has shown that their effects on vascular metrics are non-local. Therefore, directly adapting the method to generate different virtual populations may prove challenging. Indeed, as stated by Allen et al.,^69^ efficient generation of virtual populations requires knowledge of plausible ranges for the model parameters and optimizing over the set of model parameters. Reducing the number of parameters to the most influential ones appears necessary, and the sensitivity analysis presented in this study is a first step toward this goal.

At this stage, we have not considered the joint distribution of the population parameters *r_CRA_*, *MAP*, *IOP*, and *v_CRA_*. These are likely to be strongly related, and ignoring these associations may create discrepancies in the output of our model when compared to experimental data. However, these joint distributions are not readily available, to the best of our knowledge, but might be inferred from different studies in the future.

The hemodynamics model proposed in this study makes several simplifying assumptions. In particular, plasma skimming effects, which lead to non-constant hematocrit, and non-Newtonian effects, are important aspects of the hemodynamics in the microcirculation but were not incorporated in this model.^46^^,^^47^ Additionally, we assumed that there were no lateral connections between the ICP and DCP and the circulation outside the macula. This is similar to assuming that both plexi are connected in series with the SVP, which is now known to be only partially correct.^35^ Finally, the parameter *R* introduced in this model remains unknown, and its value was based on simple computation of the estimated macular blood flow. Also, uncertainty quantification showed that this parameter had a strong effect on the fraction of flow going to the macula. However, it had limited effect on the total retinal blood flow (at most 30% of variation compared to baseline for *R* varying over an order of magnitude). In future work, its influence on other hemodynamics measurements should be thoroughly evaluated.

### Applications

In future work, the validated virtual populations presented here will be used to model intra- and extravascular oxygen transport. Disease populations such as diabetic retinopathy or age-related macular degeneration, where OCTA indices have been reported to be affected by the disease, can also be generated by using population-specific distributions for the population parameters listed in Table 1 and/or by varying hyperparameters, such as θ*_min_*, which, according to the sensitivity analysis results presented earlier, can affect microvascular morphology. Additional mechanisms that may have importance in disease, such as autoregulation^21^^,^^43^ or plasma skimming,^70^ can easily be added to the current model. For example, autoregulation may be negatively affected by diabetes,^71^ while simultaneously asymmetric branching causes heterogeneous distribution of oxygen due to plasma skimming. This way, our model can be used to understand the relationship between microvasculature and pathological angiogenesis, a symptom of several blinding diseases,^11^ and provides a framework to build upon to achieve patient-specific treatment simulations.

## Conclusions

Although macular microvascular parameters serve as potent disease biomarkers, their relationship with retinal perfusion remains ambiguous. Our method establishes a versatile framework for exploring the interplay between retinal vascular structure and function. Designed to generate virtual populations from just four parameters and various quantitative OCTA-based measurements, the method can be adapted to different populations.

In our study, the model generated a population of healthy eyes, revealing robust connections between macular morphology and total retinal blood flow, independent of model parameters. The initially large hyperparameter space is effectively reduced to four hyperparameters for precise population generation.

In future work, diverse virtual populations will be created to assess model predictions in diseased maculas. This approach, complemented with hemodynamics and oxygen modeling, takes an essential step toward understanding the significance of vascular imaging biomarkers and their relation to retinal diseases.

## Supplementary Material

Supplement 1
