# Supplementary material for: Linking Vascular Structure and Function: Image-Based Virtual Populations of the Retina
Source: Invest Ophthalmol Vis Sci. 2024 Apr 29;65(4):40. doi: 10.1167/iovs.65.4.40 (PMC11059806; doi:10.1167/iovs.65.4.40)
Supplement: Supplement 1 [file iovs-65-4-40_s001.pdf]

Supplementary material S1

Statistical shape model

The curves extracted from the fundus photographs were smoothed using B-splines and their inflection points computed. For a B-spline curve $\Gamma(t) = \left(x(t), y(t)\right)$ sampled at times $t_i, i = 1, \ldots, k$, the inflection points are times t_i where $\varphi(t) = x'(t)y''(t) - x''(t)y'(t)$ changes sign, i.e., $sign\left(\varphi(t_{i-1})\right) - sign\left(\varphi(t_i)\right) \neq 0$. In this work, we used $k = 30$ and $t_i = i \times k^{-1}$ to compute the inflection points. Higher sampling rates lead to more inflections points. The start and end points of the curves along with the inflection points form the primary training data-set for the statistical shape model. Because not all segmented curves yield the same amount of inflection points, we up sample the curves by splitting the longest vessel segments in half, repeating as necessary. This results in 4 training sets, one for each arcade, with varying number of training points between sets. All shape in a training sets are aligned using the Kabsch-Umeyama algorithm [1]. Effectively, all shapes are rotated, translated and scaled as to minimise the root-mean-squared deviation between the shapes and their (aligned) mean.

For arcade vessel j, with m_j points in each shape, a data matrix $X_j \in R^{2m_j,n}$ is assembled by stacking the curves row-wise. Denoting $\left(e\middle|j, i, \lambda_{j,i}\right)$the eigenvectors and associated eigenvalues of the Pearson correlation matrix of X_j, construct $P_j \in R^{2m_j,l_j}$ by stacking the l_j eigenvectors with the largest eigenvalues column-wise. The number of modes of variations l_j may be chosen as to explain 95% of the variance in the data. Finally, denoting $\overline{x_j}$ the mean of all shapes, one can create a new, unique shape x using $x = \overline{x_j} + P_j b$ where b follows a l_j-dimensional, centered normal distribution with standard deviation $diag\left(\lambda_{j,i}\right), i = 1, \ldots, l_j$. The resulting m_j points are interpolated using centripetal Catmull-Rom splines [2] before being used in the next steps.

Sobol indices

Let $Var[Y]$ be the variance in the model's output. For $d \in N$ input parameters, $Var[Y]$ is decomposed into fractions V_u attributed to sets of inputsX_u as:

$$Var[Y] = \sum_{i=1}^{d} V_i + \sum_{1 \leq i < j \leq d} V_{i,j} + \ldots + V_{1,2,\ldots,d},$$

where

$$V_i = Var[E(Y \vee X_i)],$$

is the first order contribution of variable X_i to $Var[Y]$ and

$$V_{i,j} = Var[E(Y \vee X_i)] - V_i - V_j,$$

Is the contribution of the interactions between X_i and X_j. For higher interactions (e.g., $V_{i,j,k}$), similar expressions can be built.

In this work, we report first S_i and total S_{T_i} order indices defined as:

$$S_i = \frac{V_i}{Var[Y]},$$

$$S_{T_i} = E,$$

where $X_{\sim i}$ is the set of all input parameters excluding X_i and V_i is the first order contribution of variable X_i to $Var[Y]$ [3].

Lumen diameter may be overestimated in larger arterioles

As reported by several studies, in larger retinal arterioles (with diameter larger than 100µm), the vessel wall represents between 20 and 35% of the total vessel diameter [4-6]. In our model, we assumed that the diameters found in the literature were lumen diameter. The results of the haemodynamics simulation when reducing the diameter of larger arterioles by 20% is shown in Figure S1 and shows good agreement with experimental haemodynamics measurements.

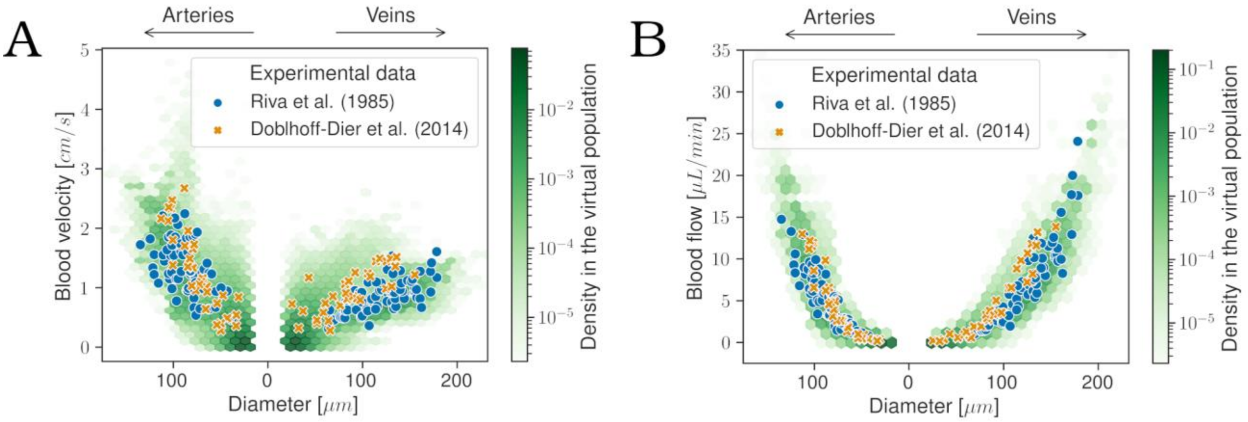

Figure S1: Blood velocity (A) and volumetric flow rates (B) distributions when accounting for a 20% overestimates of arteriole lumen.

Uncertainty quantification

Table S1 - Results from the 9 scenarios tested for uncertainty quantification where α is at baseline value.

		**Total retinal blood flow (% from change baseline)**		**Macular flow fraction (% from change baseline)**	
$\mathcal{R}$	OPP	mean	std	mean	std
$\mathbf{5 \times 10^5}$	80	13.7	1.4	11.9	8.2
	100	-7.8	1.9	12.7	8.7
	120	-29.4	2.2	12.2	8.6
$\mathbf{10^6}$	80	20	0.4	-0.2	7.3
	100	-	-	-	-
	120	-20	0.6	-0.8	8.7
$\mathbf{5 \times 10^6}$	80	38.2	2.6	-38.8	20.4
	100	22.7	3.3	-37.9	19.5
	120	7.3	3.9	-37.9	19.6

Table S2 - Pearson's R^2 scores testing linear association between $\mathcal{R}$ and OPP for the 9 scenarios tested for uncertainty quantification where α is at baseline value.

**Dependent variable**	$\mathcal{R}$			**OPP**		
**Group**	$OPP = 80$	$OPP = 100$	$OPP = 120$	$R = 5 \times 10^5$	$R = 10^6$	$R = 5 \times 10^6$
**Total retinal blood flow**	0.82	0.82	0.82	0.89	1.00	0.99
**Macular flow fraction**	0.93	0.93	0.93	0.0	0.00	0.00

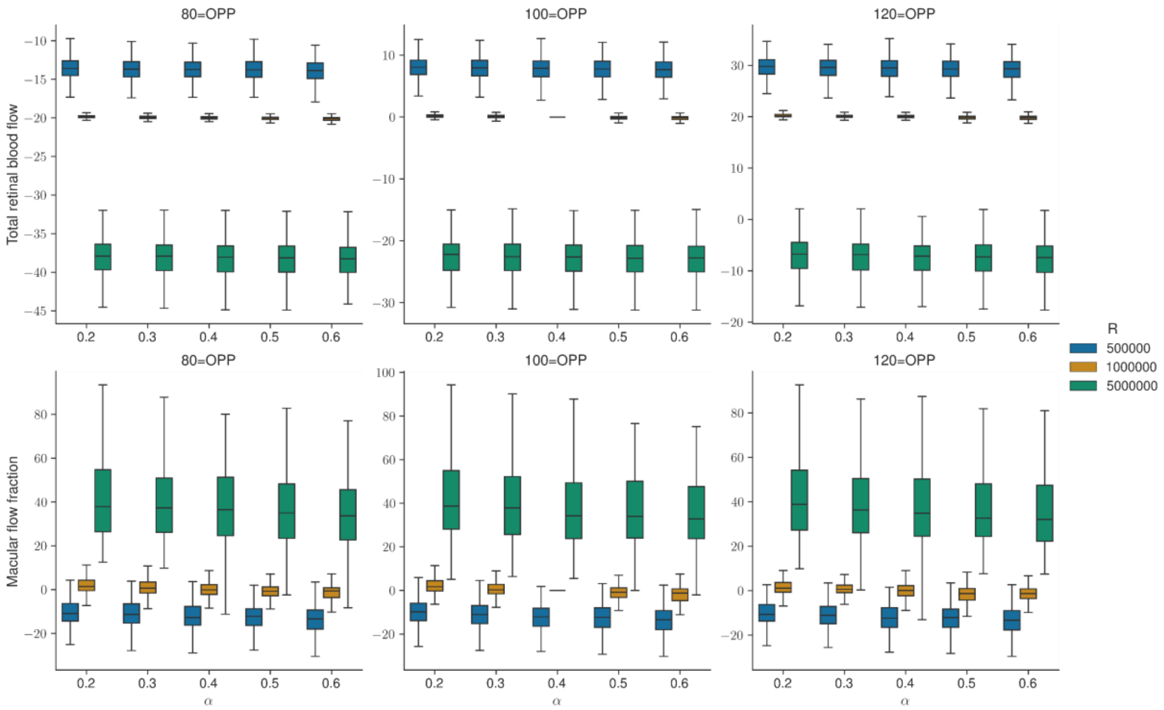

Figure S2: Effects of varying OPP, R and α on the haemodynamics outcomes.

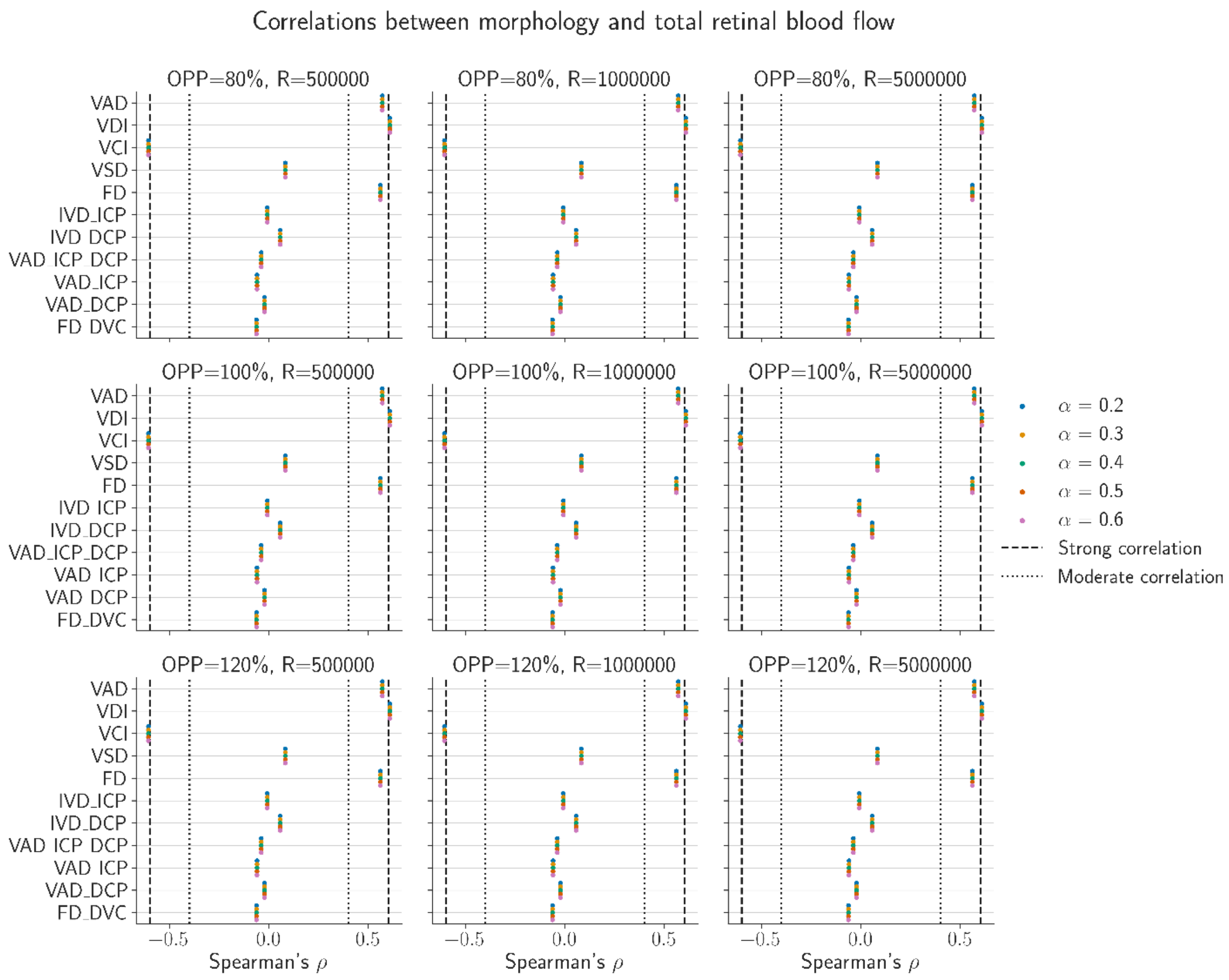

Figure S3 Effect of varying OPP, R and α on the associations between vascular structure and total retinal blood flow.

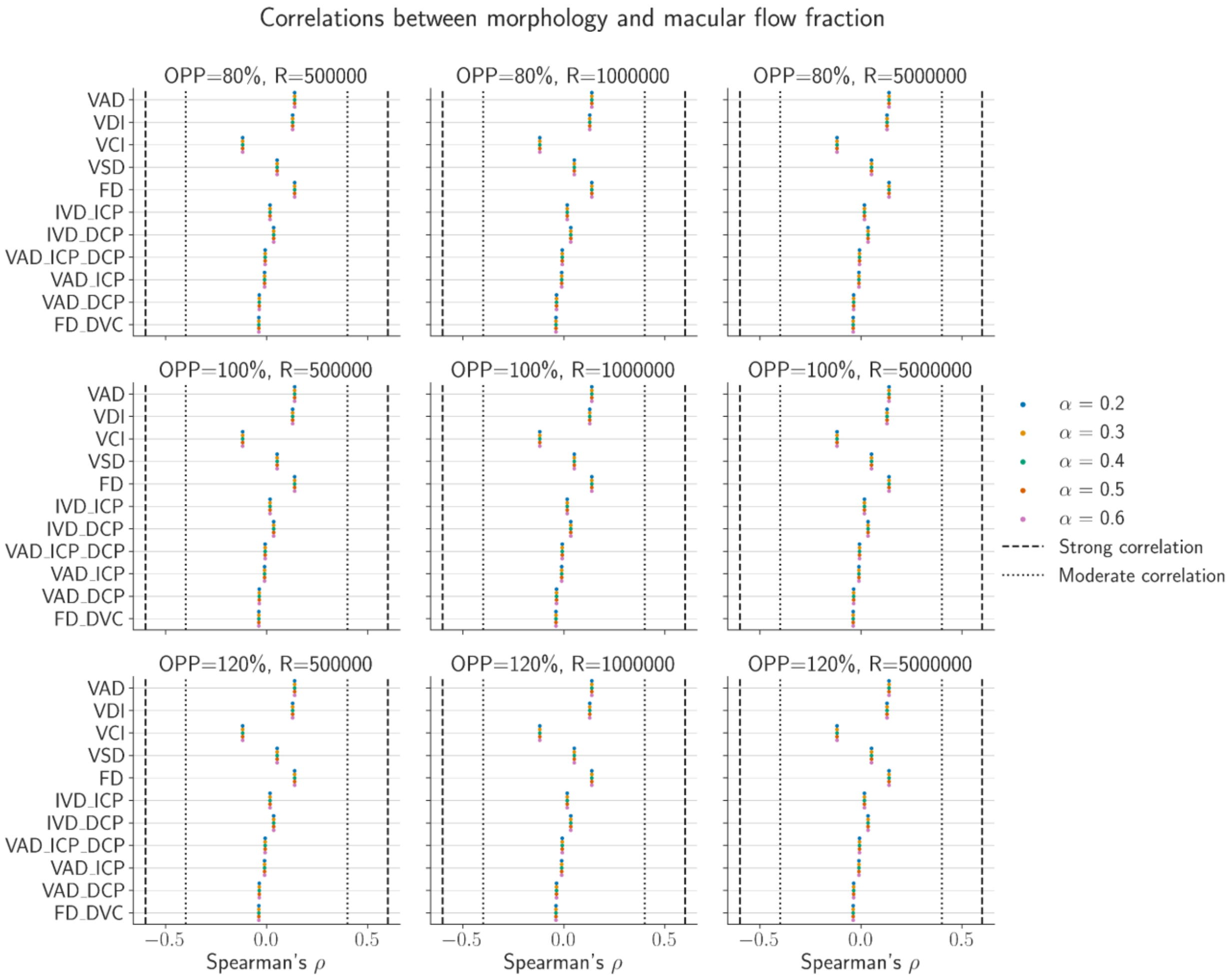

Figure S4 Effect of varying OPP, R and α on the associations between vascular structure and macular flow fraction.

References

[1] S. Umeyama. Least-squares estimation of transformation parameters between two point patterns. *IEEE Trans Pattern Anal Mach Intell*, 13(4):376–380, 1991.

[2] E. Catmull and R. Rom. A Class of Local Interpolating Splines. *In Computer Aided Geometric Design*, pages 317–326. Elsevier, 1974.

[3] A. Saltelli, Global sensitivity analysis: the primer. Wiley, Chichester, West Sussex, 2008.

[4] S. Arichika et al. Correlation of retinal arterial wall thickness with artherosclerosis predictors in type 2 diabetes without clinical retinopathy. Br J Opthalmol, 2016; 101(1):69–74.

[5] I. Damian, S. D. Nicoară. Correlations between retinal arterial morphometric parameters and neurodegeneration in patients with type 2 diabetes mellitus with no or mild diabetic retinopathy. Medicina, 2021; 57(3):244.

[6] E. Meixner, G. Michelson. Measurement of retinal wall-to-lumen ratio by adaptive optics retinal camera: a clinical research. Graefes Arch Clin Exp Ophthalmol, 2015; 253(11):1985–1995.
